# Intestinal mycobiota in health and diseases: from a disrupted equilibrium to clinical opportunities

**DOI:** 10.1186/s40168-021-01024-x

**Published:** 2021-03-14

**Authors:** Xiaoyan Wu, Yaoyao Xia, Fang He, Congrui Zhu, Wenkai Ren

**Affiliations:** 1 State Key Laboratory for Conservation and Utilization of Subtropical Agro-Bioresources, Guangdong Laboratory of Lingnan Modern Agriculture, National Engineering Research Center for Breeding Swine Industry, Guangdong Provincial Key Laboratory of Animal Nutrition Control, College of Animal Science, South China Agricultural University, Guangzhou, 510642 China; 2 College of Animal Science and Technology, Southwest University, Chongqing, 400716 China; 3 College of Veterinary Medicine, Kansas State University, Manhattan, KS USA

**Keywords:** Intestinal mycobiota, Intestinal immunity, Gut-liver axis, Gut-lung axis, Gut-brain axis

## Abstract

Bacteria, viruses, protozoa, and fungi establish a complex ecosystem in the gut. Like other microbiota, gut mycobiota plays an indispensable role in modulating intestinal physiology. Notably, the most striking characteristics of intestinal fungi are their extraintestinal functions. Here, we provide a comprehensive review of the importance of gut fungi in the regulation of intestinal, pulmonary, hepatic, renal, pancreatic, and brain functions, and we present possible opportunities for the application of gut mycobiota to alleviate/treat human diseases.

**Video Abstract**.

**Supplementary Information:**

The online version contains supplementary material available at 10.1186/s40168-021-01024-x.

## Introduction

Intestinal tract is home to bacteria, archaea, fungi, viruses, and protozoa, which mutually interact with the host [1, 2]. In general, investigation of intestinal commensal-host symbiosis places emphasis on gut bacteria while neglecting gut fungi due to their lower abundance (0.01–0.1% of gut microbiome) [1, 3, 4]. For example, in the period of 2008–2018, there have been almost 100 times more peer-reviewed publications on microbiota than on mycobiota [5].

Commensal fungi exist in various body parts (e.g., oral cavity, gastrointestinal tract, and vagina) and sustain a relative stable colonization [6, 7]. Invasion by exogenous pathogenic fungi (e.g., inhaled environmental fungi) can disrupt the fungal homeostasis in the lungs; uncontrollable transfer of commensal fungi in immune incompetent individuals also leads to systemic disequilibrium [3, 8]. Thus, fungi such as *Candida albicans* [9], *Zygomycetes*, *Fusarium*, *Acremonium*, *Trichosporon*, and *Penicillium marneffei* [10] are associated with the occurrence of hospital-acquired and/or intercurrent fungal infections while the individuals are immunocompromised. In addition to causing infections, fungi affect the host immune responses to other stimuli both locally and distally [11, 12]. For example, recognition of β-1,2-mannans (a component of fungal cell wall) by galectin-3 [13] enhances toll-like receptor (TLR) 2- and TLR4-mediated immune responses in mouse primary splenocytes and human THP-1 cells [14, 15]. Thus, fungi are associated with the health and disease of the host. Notably, the effects of bacteria and fungi on the immune system are very similar, and intestinal bacteria could influence intestinal fungi; thus, the interaction between gut fungi and bacteria should not be neglected. For example, *Escherichia coli* super-infection promotes *C. albicans* virulence under certain circumstances [16], and commensal bacterial in the intestine of adult mice, especially *Bacteroidetes* and *Firmicutes* species, are major resisters for *C. albicans* colonization [17].

Recent evidence of a significant influence of gut mycobiota on host’s health has stimulated further research on gut mycobiota. Notably, despite relatively small number of gut fungi, they profoundly affect nutrition, metabolism, and immunity in the intestine [1, 2, 18–20]. Not only do intestinal fungi shape the functions of the gut, but they also affect the physiological functions of other crucial extraintestinal organs, such as the liver, lung, and brain [1]. Here, we summarize the current knowledge about gut mycobiota, including the methodologies for studying gut fungi, colonization, and composition of gut mycobiota, and the affecting factors. We also highlight the importance of gut fungi on the intestine and intestinal-associated distal targets, including gut-lung axis, gut-liver axis, gut-brain axis, and possibly gut-kidney axis and gut-pancreas axis. Multiple targets of gut fungi may offer new possibilities for diagnosis and treatment of various diseases.

## Methodologies for studying gut fungi

There is still relatively poor understanding of the influence of gut mycobiota on host’s health and disease. One of main reason is that fungi have been traditionally investigated through culture-dependent methods, which largely limited the in-depth understanding of the fungal microbiota. Fortunately, the study of fungi populations benefited from deep-sequencing technologies and bioinformatics analysis developed for bacteria [e.g., next-generation sequencing (NGS)]. In this section, we briefly introduce the methodologies for studying gut mycobiome.

### Culture-dependent methods

Traditionally, fungal diversity was assessed using culture-dependent methods, which highly depended on the culture medium. For example, Sabouraud dextrose agar (SDA) is a commonly used medium for fungi especially for filamentous fungi [21, 22], while blood agar (BA) and chocolate agar (CA) are used for fungi isolated from mycotic keratitis patients [21]. CHROMagarTM Candida medium has been used for *Candida* separation [23, 24], whereas ID fungi plate culture with matrix-assisted laser desorption ionization time of flight mass spectrometry (MALDI-TOF MS) is suitable for pathogenic filamentous fungi [25]. Obviously, cultivation of fungi is the most direct method with visualization of fungal morphology and colony color. However, there are some limitations in its application. (1) During cultivation, the environmental fungi (e.g., *Aspergillus*) easily affect the precision of results, and even slight differences in culture process lead to the change in fungi morphology [25]. (2) It is difficult to distinguish specific species and genus of similarly looking fungi even by microscope, and some fungi cannot be cultured in the existing culture modes [26]. (3) Culture methods are very time-consuming and not adaptable to high-throughput analysis. Fortunately, some new culture-dependent approaches (e.g., culturomics or combination of culture and sequencing) are now used for fungus characterization [27].

### Culture-independent methods

Recent advances in deep-sequencing technologies and bioinformatics analysis, which are based on the analysis of genomic DNA, have shed light on the complexity of the gut fungal communities. Interestingly, these methods could also be applied to a single fungus isolated from culture-dependent methods. Generally, many culture-independent methods have been developed, such as sequencing for 18s ribosomal DNA and internal transcribed spacer regions (ITS, 1 and 2) [28], denaturing gradient gel electrophoresis (DGGE) [7], amplified fragment length polymorphism fingerprinting (AFLP) [29], restriction fragment length polymorphoresis (RFLP), oligonucleotide fingerprinting of ribosomal RNA genes (OFRG) [30], and high-throughput sequence technology (HTST) [31]. Of note, the factors affecting fungi identification with culture-independent methods include DNA extraction (quality and quantity), primer selection (e.g., preferential ITS fragment), and bioinformatics pipelines (e.g., assignment algorithms) and databases (available or not) [32].

Overall, there is no consensus on the optimal methodology for characterizing gut mycobiome, and the available results vary depending on the chosen analytic method [33, 34], indicating that the methods selected for fungal analysis should be noted when comparing the results on intestinal fungi between the studies.

## Colonization and composition of gut mycobiota

With the recent development of methodology, we have reached preliminary understanding of the colonization and composition of intestinal fungi. Gut colonization of mycobiota in humans has been established at 10 days after birth [35]. In the neonatal gut, the alpha diversity of fungi consistently increases from birth to 2 years of age, while the beta diversity reaches to the peak in a 10-day infant [35]. However, given the limitation of samples and methods for fungal detection, we cannot rule out the possibility that the colonization of gut fungi starts at the time of birth or even earlier (e.g., amniotic fluid exposure, umbilical substance exchange with mother [36]). Recently, fungi have been found in the first-pass meconium, suggesting that colonization of gut mycobiota starts at birth [37]. Nevertheless, due to the lack of direct evidence and low biomass of meconium, it is difficult to ignore the effects of environmental factors. New models that could mimic the entire pregnancy and advanced detection techniques are urgently needed for more convincing conclusions.

Similar with the gut bacteriota, there are temporal and spatial changes in the distribution and composition of gut mycobiota. It has been reported that in 10-day- to 3-month-old infants, *Debaryomyces hansenii* and *Rhodotorula mucilaginosa* are the most abundant fungi, while after 1–2 years, the composition of gut mycobiota changes, where *S. cerevisiae* becomes the most abundant fungus while the *Candida* spp. start to decrease [35]. Moreover, *Aspergillus penicillioides* is not detected in infants aged 10 days to 3 months, but could be detected in 1- to 2-year-old infants [35]. Intriguingly, *Cystofilobasidium* sp., *Ascomycota* sp., and *Monographella* sp. are only detected in 1- to 2-year-old offspring [35]. Afterwards, *Ascomycota*, *Zygomycota*, and *Basidiomycota* phyla dominate in healthy intestinal mycobiota [38], and *Candida* is the most prevalent and abundant fungi dwelling in gastrointestinal tract and other mucosal surfaces in humans and several other animals [2, 3, 39], while other genera include *Pichia, Saccharomyces, Cladosporium, Malassezia, Aspergillus, Trichosporon, Penicillium*, and *Mucor* [2]. Overall, the characterization of colonization and composition of intestinal fungi is still in its infancy.

## Factors affecting the composition of gut mycobiota

Of note, similar like gut bacteria [36], gut fungi colonize neonatal gut instantly after birth, while the composition could be affected by delivery mode, gestational age at birth, infant feeding mode, maternal diet, environment, and host genetics (Table 1).

**Table 1:** Factors affecting the composition of gut mycobiota

Factors			Composition of gut mycobiota	References
Delivery method		Natural birth	Fungi from mother’s genital tract ↑ Russulales ↑	[37, 40–42]
		C-section	Fungi from maternal skin and surroundings ↑ Saccharomycetales ↑	
Gestational age		Preterm infants	Fungal diversity ↓ Saccharomycetales ↑ *Candida* ↑	[37]
		Term infants	Polyporales ↑ Russulales ↑ *Stereum* ↑ *Malassezia* ↑	
Environment		Mice from Jackson Laboratory’s & Services (JAX)	Basidiomycota ↑	[18, 43]
		Mice from Weill Cornell Medicine (WCM-CE)	Ascomycota ↑	
		SPF mice "rewilded" into the wild	*Candida* ↑ *Aspergillus* ↑	
Season		Spring	Sclerotiniaceae ↑ Nectriaceae ↑	[19]
		Summer	Trichocomaceae ↑	
		Autumn	Wallemiaceae ↑ Hypocreaceae ↑	
		Winter	Devriesia ↑	
Diet and nutrition	Nutrition	Pistachio and almond	*Penicillium* spp*.* ↓ *Candida* spp*.* ↓	[1, 27, 44–50]
		Carbohydrate-rich diet	*Candida* ↑	
		High-fat diet	*S. cerevisiae* ↓	
		Protein-rich diet	*Methanobrevibacter* ↓ *Candida* ↓	
		2-hydroxyisocaproic acid (leucine derivative)	*Candida* ↓ *Aspergillus* ↓	
	Microbial metabolites of nutrients	Short chain fatty acid (SCFAs)	*Aspergillus* ↓ *Metschnikowia* ↓	
		Acetate	*Tomentella* ↑	
		Acetate and propionate	*Nephroma* ↓ *Taiwanofungus* ↓	
		Butyrate and total SCFAs	*Tomentella* ↑	
		Propionate	*Loreleia* ↑	
Gender		Female	*Mycosphaerellaceae* ↑	[19]
		Male	*Ascomycota* ↑ *Tetraplosphaeriaceae* ↑	
Metabolic disorder		Obese	Yeast fungi ↑	[27]
		Eutrophic	Filamentous fungi ↑	
Maternal antibiotic exposure			Saccharomycetales ↑	[37]
Species			Gut *Candida* spp. only found in mammalian	[44, 49]
			Chenghua, Yorkshire, and Tibetan pigs have different fungal abundance	

“↑” indicates increase and “↓” indicates decrease

### Delivery mode and maternal probiotic exposure

For neonates, the mother is one of the main factors affecting their gut fungal composition. Namely, healthy gut mycobiota colonization at birth is affected by delivery mode (vaginal versus cesarean) [40]. Naturally born infants are more likely to get fungi (e.g., *Candida albicans*) from maternal genital tract, whereas infants born after cesarean delivery are more likely to get fungi from maternal skin and surroundings [41]. Apart from the mode of delivery, gestational age and maternal probiotic exposure also determine gut mycobiota composition in infants [37]. However, in 298 pairs of mother-offspring subjects, Schei et al. reported that the type of delivery and/or use of infant antibiotic or maternal probiotic had little effect on the operational taxonomic units (OTUs) abundance of gut fungi [35]. This discrepancy may stem from the samples analyzed since they selected mother-offspring pairs who all participated in a single study.

### Diet and nutrition

Diet is one of the determinants in shaping gut mycobiota composition. It has even been suggested that the source of fungi in the oral cavity and diet can explain all the fungi present in the feces of healthy subjects, suggesting the great influence of diet on intestinal fungal composition [51]. Interestingly, Western diet has a high risk of triggering metabolic syndrome due to high levels of fats and carbohydrates, and it has been shown to induce the alterations of intestinal fungal structure of human [52]. For example, diet with high amounts of fat decreases *S. cerevisiae* abundance in the gut of mice [1]. The abundance of *Metschnikowia*, *Tomentella*, and *Loreleia* correlates with short chain fat acids (SCFAs) in pigs [44], and SCFAs are negatively associated with the intestinal abundance of *Aspergillus* in human [45]. Moreover, carbohydrate-rich diet correlates positively with the abundance of gut *Candida* [46]. Likewise, diets with high levels of protein correlate negatively with the abundance of *Methanobrevibacter* and *Candida* in healthy volunteers [45]. Interestingly, *S. cerevisiae* have amino acid transporters, and some amino acids, such as gamma-aminobutyric acid (GABA) and citrulline, are important nitrogen sources for *S. cerevisiae*; thus, dietary amino acids probably have profound impact on gut mycobiota composition [53]. Furthermore, 2-hydroxyisocaproic acid, a metabolic by-product of leucine, is fungicidal at 72 mg/mL and fungistatic at a lower concentration, which could inhibit *Candida* hyphal formation [47]. Particular chemical compounds in human diet, such as phytochemicals from pistachio and almond, also negatively correlate with the abundance of *Penicillium* spp. and *Candida* spp. [48]. Taken together, it is important to examine whether diet control has potential to alleviate fungal infections. If this strategy works, it could be particularly beneficial for immunosuppressed patients to prevent and control secondary infections.

### Other factors

Environmental fungi pass through the respiratory and gastrointestinal tract, and they can be a source of transitory colonization of gut mycobiota [5]. Facility environments also are capable of triggering changes in gut mycobiota. Interestingly, C57BL/6J mice acquired from Jackson Laboratory’s Mice & Services (JAX) and those bred at Weill Cornell Medicine (WCM-CE) differ in the composition of gut mycobiota and are dominated by *Basidiomycota* and *Ascomycota*, respectively [18]. Moreover, when SPF mice are “rewilded” into the wild condition, they experience a significant increase in intestinal fungi [43]. Season is another factor altering gut mycobiota, especially alpha diversity of fungi [19]. Gender and metabolic disorder (eutrophic, overweight, and obesity) shift gut mycobiota as well. For instance, female Tibetan macaques have different mycobiota compared with male Tibetan macaques [19]. Increased yeast is observed in obese human individuals, whereas eutrophic and overweight human individuals have more filamentous fungi [27]. Gut *Candida* spp. is found exclusively in mammalian species [49]. Therefore, to some extent, gut mycobiota probably varies by species or genotypes [44]. Collectively, gut mycobiota is affected by both internal and external factors (Table 1).

It should be noted that these factors are not independent. Namely, season and environment are associated with available food that mammals live on, especially for wild animals [19]. The environment also affects host’s exposure to the potential pathogenic or non-pathogenic microorganisms [43]. Moreover, different breeds always have different genetic background, not to mention food available to them [44]. Because offspring at 10 days and 3 months are almost exclusively breast-fed, their gut mycobiota are dominated by *Debaryomyces hansenii* from breast milk [35]. Consequently, the change in intestinal fungal communities from *D. hansenii* to *S. cerevisiae* has been observed to occur with the shift in diet from breastfeeding or formula milk to solid food [54]. Besides, with further growth, infants are subjected to diverse diets, exposed to complex environments and stimulus such as drugs and sexual hormones [27]. Therefore, these factors must be recognized in a systematic and interconnected manner, and it is not appropriate to overemphasize the unique roles of each factor alone.

## Cross-talk between gut mycobiota and host immunity

**Fig. 1:**
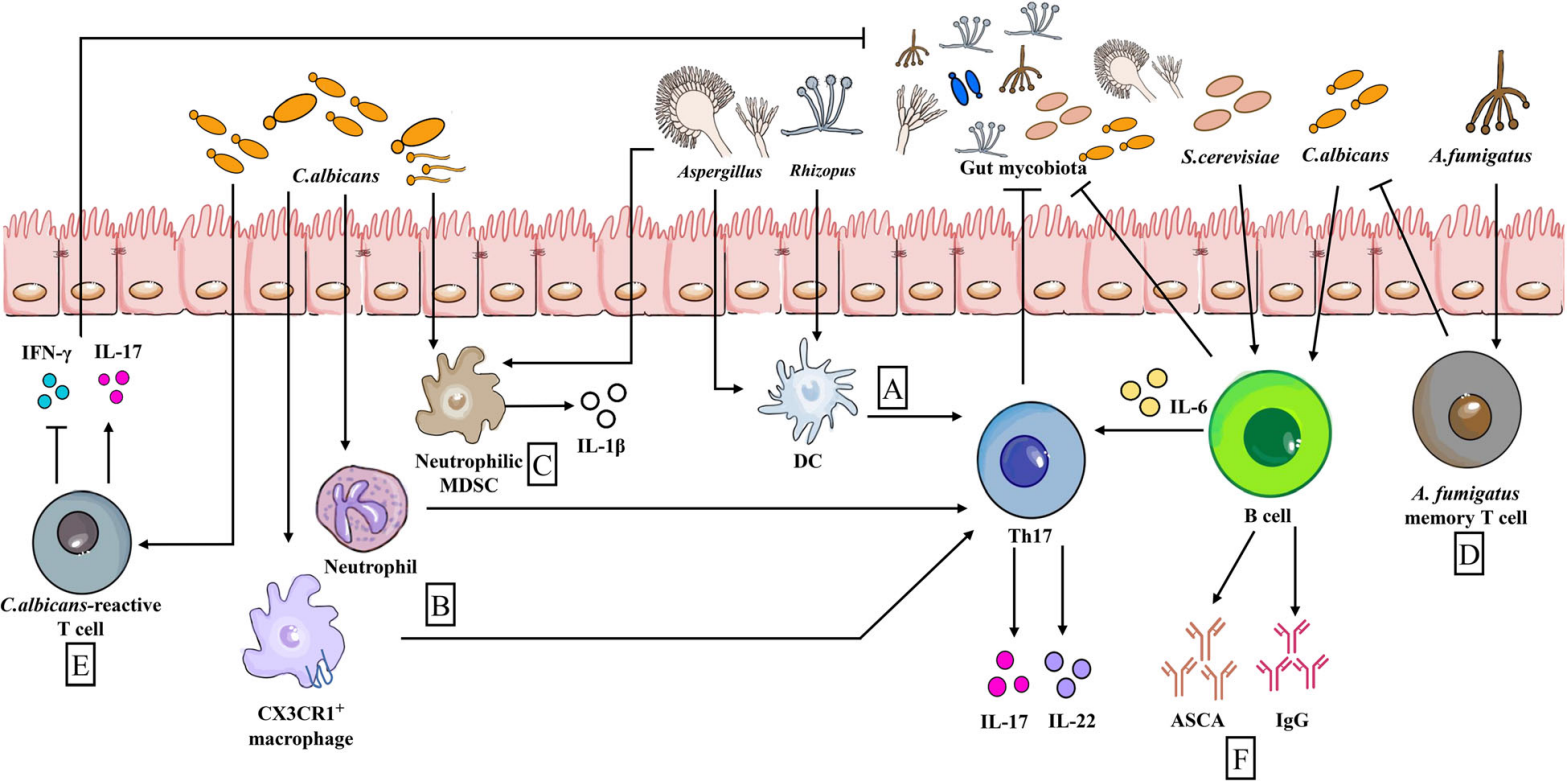
Influence of gut mycobiota on intestinal immunity. (**a**) When triggered by *Aspergillus* and *Rhizopus*, DCs promote Th17 responses. (**b**) CX3CR1^+^ macrophages and neutrophils are beneficial for early *Candida* control by inducing robust Th17 responses with production of high levels of IL-17 and IL-22. (**c**) Likewise, invading *C. albicans* and *Aspergillus* trigger neutrophilic MDSCs and production of IL-1β. (**d**) *A. fumigatus* memory T cells show cross-reactivity with *C. albicans* because they have a shared TCR sequence. (**e**) Likewise, *C. albicans*-reactive T cells cross-react with other gut fungi by producing IL-17. (**f**) ASCA and IgG (stimulated by *C. albicans* or *S. cerevisiae*) from B cells are able to anti-intestinal fungus. Besides, B cells also participate in anti-fungal immunity in an antibody-independent manner, and IL-6 from B cells enhances anti-fungal Th17 responses. DCs, Dendritic cells; MDSCs, myeloid-derived suppressor cells; IgG, Immunoglobulin G; TCR, T cell receptor; ASCA, Anti-*Saccharomyces cerevisiae* antibody. Arrows represent activation and horizontal lines represent suppression

Among the different kingdoms represented in the gut microbiota, the eubacteria and their effect on the host have been studied most extensively. Similar to gut bacteria, gut fungi are highly pleiotropic and regulate various physiological functions in the host. Among them, several fields deserve particular emphasis, such as modulation of host’s metabolism (e.g., purine metabolism) [55], control of aging and disease progression [40, 56], and, with particular relevance to this article, immune homeostasis [1, 57]. However, reports on intestinal mycobiota and host immunity are fragmented, and whether or how most intestinal commensal fungi interact with the host immune system is still the topic of investigation. The current understanding of the influence of intestinal mycobiota on the pathways and cellular networks of immune responses has been reviewed recently [1]; thus, here, we present a brief summary (Fig. 1).

The cell wall of some commensal fungi, such as *S. cerevisiae* and *C. albicans,* contains various ligands (e.g., β-glucans and chitin) for the receptors of innate immune cells (see review by Wheeler et al. [58] for more information about immunological recognition of fungus). Generally, as important pathogen recognition receptors (PRRs), C-type lectin receptors (CLRs) such as Dectin-1, -2, and Mincle are mainly expressed in dendritic cells (DCs) and macrophages to recognize commensal fungi [59]. Upon ligation of CLRs, nuclear factor-kappa B (NF-κB) and mitogen-activated protein kinase (MAPKs) are activated via CARD9-BCL10-MALT1 complex, which modulates pro-inflammatory cytokines and ROS production, finally restricting the growth of fungi [59]. Dectin-1 is also related to Syk recruitment and PKCδ activation [59]. Thus, lack of Dectin-1, Dectin-3, or CARD9 may result in dysbiosis of gut fungi [60]. Besides CLRs, TLRs function as direct recognizers of fungi; however, because of heterogeneous morphologies, fungi express different ligands and induce multitudinous immune responses [61]. For example, upon *C. albicans* colonization, TLR1^−/−^ and TLR2^−/−^ mice experience aggravated intestinal colitis, whereas in TLR6^−/−^ mice *C. albicans* is nearly cleared [62].

Neutrophils could release nuclear DNA to form a network [named neutrophil extracellular traps (NET)] for trapping and killing extracellular pathogenic microorganisms [63]. Intriguingly, it has been found that the Syk-PKC-ROS cascade is activated by opsonized *C. albicans* through complement receptor 3 recognition, causing NADPH oxidase-dependent NET [64]. However, unopsonized *C. albicans* recognition by Dectin-2 activates Syk-Ca2^+^-PKCδ-PAD4 signaling pathway, triggering NADPH oxidase-independent NET [65]. Notably, PAD4 inhibition (GSK484), neutrophil elastase (NE) inhibition (Sivelestat), and/or Dectin-2 deficiency affect PAD4 activation, NE nuclear translocation, and histone H3-containing NET initiation result in increased renal fungal burden, suggesting that NET is involved in inhibiting remote fungal transmission [65]. Moreover, NET functions as a part of innate immune defense against fungal pathogens (e.g., *Aspergillus* spp*.* and *Paracoccidioides* spp*.*), which has been comprehensively discussed elsewhere [66].

Usually, intestinal bacteria induce the differentiation of tolerogenic DCs to induce Foxp3^+^ Treg cells [67]; however, invasion by opportunistic pathogenic fungi (e.g., *Aspergillus* or *Rhizopus*) induces DCs to promote Th17 responses [68]. There are various subtypes of macrophages, like CX3CR1^+^ and CD103^+^ macrophages [69]. After recognition of commensal and/or pathogenic mycobiota (e.g., *Candida* [70]) via CLRs, CX3CR^+^ macrophages participate in inducing Th17 responses to resist infection [71]. CX3CR1^+^ macrophages are needed for early *Candida* control by restraining caspase-dependent apoptosis and promoting Akt phosphorylation [71, 72]. In addition, a polymorphism mutation of *CX3CR1* gene impairs generation of IgG in recognizing Ascomycota phylum (e.g., *C. albicans*, *Pichia kudrazevii, S. cerevisiae,* and *Aspergillus amstellodamii*), whereas recognition of flagellin remains unaffected [71], implying that CX3CR1^+^ macrophages have unique response modes to the gut fungi. The status of CD103^+^ macrophages in initiating anti-fungal responses needs further study. Of note, *Candida tropicalis* colonization drives CD103^+^ RALDH^+^ (retinaldehyde dehydrogenase) DCs migration and production of retinoic acid (RA) [73], which affects anti-inflammatory responses of intestinal macrophages, and establishes connection between intestinal fungi and peripheral immune system. Unfortunately, *C. tropicalis* is not common in the human gut [73, 74], so the role of CD103^+^ macrophages in human anti-fungal responses remains to be explored.

Gut fungi shape the fate decision of innate immune cells and also of T cells and B cells. Regardless of the form (yeast or hyphae), *C. albicans* uniquely stimulates robust Th17 responses [12]. Colonization of *C. albicans* drives robust fungal-reactive specific Th17 cells and circulating IL-17-reactive neutrophils [75], which was observed in mesenteric lymph nodes and colon [71]. Such immune response induced by *Candida* colonization blocks the invasion by exogenous *Candida* and reduces invasion by *S. aureus* [75]. Interestingly, memory T cells for *A. fumigatus* show cross-reactivity with *C. albicans* because they have a shared T cell receptor (TCR) sequence [12]. Furthermore, *C. albicans* cross-reactive T cells perform a variety of responses to other fungi [12]. B cells produce highly effective antibodies against the invading pathogenic fungi. For example, anti-Saccharomyces cerevisiae antibody (ASCA ) induced by *S. cerevisiae* or *C. albicans* is necessary for anti-intestinal fungal infection [71], especially IgG is probably involved in recognizing and resisting intestinal *C. albicans*. Interestingly, B cells also participate in anti-fungal immunity through antibody-independent manner by inducing antigen specific T cells and especially anti-fungal Th17 responses [76].

However, we are still lacking sufficient data about the influences of intestinal mycobiota on other immune cells, such as natural killer (NK) cells, mast cells, and innate lymphoid cells (ILCs), as well as on non-immune cells, such as intestinal epithelial cells. Intestinal epithelial cells are vital barriers [77–79], and the first and essential step in *Candida* infection involves adherence to epithelial cells, followed by invasion of this barrier primarily by active penetration [80, 81]. Although it has been recently reported that distal colon “balloon-like” protrusions (BLPs)^+^ macrophages could prevent the absorption of fungal toxins, protecting the barrier integrity [82], the influence of intestinal mycobiota on the cellular composition of intestine needs further investigation. In particular, some metabolites (e.g., indoles) from intestinal microbiota highly shape the cell fate of intestinal cells [83, 84]. More importantly, at present, the research on the influence of intestinal mycobiota to host immunity is still in its early stages. Studying gut mycobiota as a collective unit contributes to a better understanding of its deep role in the host, such as the significance of change in their composition and colonization over time, and the possibility of improving host immunity through external intervention to artificially regulate the intestinal fungal community.

Besides the direct recognition by host cells, intestinal mycobiota produces some pleiotropic substances, including candidalysin and farnesol, to regulate host immune responses (Table 2). Candidalysin, firstly detected in 2016 from *Candida albicans* [111], affects protective immune responses and systematic infection [98, 99]. Interestingly, candidalysin utilizes NLRP3 inflammasome-dependent cytolysis to evade phagocytic clearance [100, 101]. In addition, it is positively associated with severity of ethanol-associated hepatitis [102]. Farnesol, one of the Candida-secreted quorum-sensing molecules (QSMs) [112], acts as vital virulence factor to impair the ability of immature DCs (iDC) to induce T cell differentiation and expression of pro-inflammatory cytokines, thereby, affecting pro-inflammatory and Th1 responses [85]. Notably, fungi also secrete prostaglandins (PGs) or convert exogenous arachidonic acid (AA) into PGs [103–105] to affect the functions of phagocytes, which contributes to continuous colonization of *C. albicans* [103, 106–108]. Moreover, fungal oxylipins are vital factors in modulating immune responses [109, 110].

**Table 2:** Fungi-derived compounds and their functions

Fungi-derived compounds		Functions	References
Quorum-sensing molecules	Farnesol	Filamentous growth and formation ↓ *C. albicans* biofilm formation ↓	[85–91]
		Drug-resistance ↓ Synergy with fluconazole, amphotericin B or micafungin, anti-fungal AMP peptidomimetics, chitosan (CS), and cysteine protease metacaspase	
	Tyrosol	*C. albicans* biofilm formation ↓	
Fungal secondary metabolites		Source of antibiotics and immunosuppressant drugs Hepatotoxicity and/or nephrotoxicity	[54, 92, 93]
Fungi-derived extractives	(1,3)/(1,6)-β-glucan	Obesity property ↓	[94–97]
	Pyronepolyene C-glucoside iso-D8646-2-6	Influenza A virus (H1N1) infection ↓	
	Xanthones	Virus infection (H1N1, simplex virus types 1 and 2) ↓	
	Some mangrove-associated or soil-associated fungus-derived compounds	Virus infection ↓	
Other compounds	Candidalysin	Systematic infection ↑ Phagocytic clearance ↓ Severity of ethanol-associated hepatitis ↑	[98–110]
	Prostaglandins E2 (PGE2)	Biofilm formation ↑ Yeast to hyphae transition ↑ *C. albicans* clearance by phagocytes ↓	
	Oxylipins	Modulating immune responses	

“↑” indicates increase and “↓” indicates decrease

In addition to the aforementioned fungal components and fungal metabolites, extracts, and secretions, secondary metabolites are also responsible for influencing host homeostasis (Table 2) [113]. Various clusters of genes for production of secondary metabolites indicate that fungi operate complex pathways to produce secondary metabolites, which have huge structural and functional diversity (see a review by Macheleidt et al. [92]). Notably, fungal secondary metabolites have limitless potential in fungal diseases and synthetic biology; they are also prolific sources of antibiotics (e.g., penicillins and cephalosporins) and immunosuppressant drugs (reviewed in [93]) for their ability in targeting or interfering with fungi and/or bacteria. However, some of the fungal secondary metabolites, including aflatoxin and citritin, have strong hepatotoxicity and/or nephrotoxicity [54].

## Gut mycobiota in intestinal and extraintestinal diseases

As Wheeler et al. determined that disturbance of intestinal fungi with prolonged anti-fungal treatment in mice deteriorated colitis and even worsens allergic airway disease [114], imbalance in the gut mycobiota composition may be associated with intestinal and also extraintestinal diseases. Although the complete intestinal targets for gut fungi have not been determined completely, it is clear that gut fungi affect more than the intestinal tract and lungs. We review the related investigations and propose several possible fungal targets: intestinal tract, lung, liver, kidney, pancreas, and brain. Within each niche, dysbiosis of gut mycobiota may be related to the progression of certain diseases (Table 3). Whenever available, we also highlight the major findings on the potential mechanisms by which gut mycobiota affects disease status.

**Table 3:** Association of enteral and parenteral diseases with gut mycobiota

Targets		Diseases	Related fungi	Reference
Intestinal tract		Inflammatory bowel disease (IBD)	Basidiomycota ↑ Ascomycota ↓ *Candida* ↑ *S. cerevisiae* ↓	[115–118]
		Celiac disease	*Candida*↑	[119, 120]
		Colon cancer	*C. tropicalis* ↑	[121, 122]
Extra-intertinal tract	Lung	Fluconazole induced Allergic airway disease (AAD)	*Candida* ↓ Aspergillus ↑ Wallemia ↑ Epicoccum ↑	[11, 114]
		Pulmonary infection	*Histoplasma capsulatum*	[123]
	Liver	Cirrhosis	Fungal detection ↑	[124]
	Kidney (possible)	Sepsis	*C. albicans*	[125, 126]
	Pancreas	Pancreatic ductal adenocarcinoma (PDA)	*Malassezia* ↑	[127]
	Brain	Multiple sclerosis (MS)	*Candida*	[128]
		Schizophrenia (SCs)	*Chaetomium* ↑	[129]

“↑” indicates increase and “↓” indicates decrease

### Gut mycobiota and intestinal diseases

Specifically, healthy gut mycobiota is dominated by several abundant commensal fungi in various species (e.g., *Candida tropicalis* and *S. cerevisiae* in C57BL/6 mice [130], and *Saccharomyces*, *Malassezia*, and *Candida* in humans [6, 74]). Notably, gut mycobiome could be the reservoir of opportunistic pathogens, although some commensal fungi (e.g., *Candida albicans*) are recognized as the true symbionts [3, 8, 39, 131–133]. Inflammatory bowel disease (IBD), including ulcerative disease (UC) and Crohn’s disease (CD), is associated with dysbiosis of gut mycobiota. Fecal samples from children with IBD show lower fungal diversity compared with healthy children, while *Candida* genus is twofold more abundant in IBD samples (72.9%) than in healthy controls (32.9%) [115]. These results are in agreement with previous reports of UC- and CD-associated intestinal colonization by *C. albicans* in human [116, 117]. Moreover, *C. albicans* increases in IBD flares compared with remissions [118]. Recently, Limon et al. have reported that *Malassezia restricta*, the common skin resident fungus, is linked to the pathogenesis of CD, especially in patients harboring the IBD-linked polymorphism in CARD9 (S12N), and that the colonization of *M. restricta* exacerbates disease severity in DSS-induced colitis in murine models [134]. This study suggests that, besides well-known *C. albicans*, other identified fungi can be involved in the development of IBD, and that genetic factors, especially CARD9 polymorphisms, are important in defining the inflammatory responses to colonization. Also, the ratio of fungi-to-bacteria diversity increases in CD samples, suggesting the intestinal environment of CD patients is probably more suitable for fungal colonization [114, 118, 135]. Collectively, the alterations in gut environment during IBD are associated with changes in various fungi and bacteria and induce changes in fungal-bacterial relationship [118, 136]. However, the ultimate destiny of host IBD is not only related to the bacterial and/or fungal communities that affect intestinal homeostasis, but the host immunity plays a decisive role [71, 137]. More importantly, the aforementioned results focused on the characterization of fungal communities in CD and/or a mixed UC and CD patients or experimental models; further independent studies about the variation of gut fungal communities in UC patients or experimental models are needed. Interestingly, *S. cerevisiae* is a beneficial fungus that has capacity to ameliorate gastroenteritis [3, 138], mitigate adherent-invasive *Escherichia coli* (AIEC)-induced colitis in CEACAM6 (carcinoembryonic antigen-related cell adhesion molecule 6) -expressing mice [139], and relieve abdominal pain in irritable bowel syndrome (IBS) in human subjects [140]. Therefore, the modulation of intestinal mycobiota may be a potential target for treating IBD. The cause-effect relationship between intestinal commensal fungi and IBD should be further investigated. Moreover, it is still unclear whether gut fungi affect IBD progression by interacting with gut bacteria.

In addition to IBD, celiac disease (CeD) has been suggested to be associated with disorders of intestinal mycobiota. The typical sign of CeD is reversible mucosal atrophy of the small-bowel, and atypical clinical symptoms often result in a missed diagnosis of CeD, which leads to increased disease severity [141]. *Candida* is related to the pathogenesis of CeD [119]. Specifically, the association between *C. albicans* and CeD began with the hypothesis that *C. albican*s virulence factor-hyphal wall protein 1 (HWP1), which is identical or highly homologous with the CeD associated-α and γ gliadin of T cell antigen epitopes, and serves as a transglutaminase (TG) substrate, assisting in the production of autoreactive antibodies. Therefore, *C. albicans* may be one of the underlying reasons for CeD [119]. Later, by using antigen-antibody reaction and microchip analysis, both CeD group and *C. albicans* infection (CI) group expressed high anti-HWP1, anti-gliadin antibody and anti-transglutaminase (anti-TG) IgA antibody, but CeD had a higher reaction to HMP1 [142]. However, more evidence is needed to establish the involvement of *C. albicans* in CeD.

Moreover, colon cancer is also associated with dysbiosis of intestinal fungi, especially a significant increase of *C. tropicalis* in CARD9^−/−^ mice [121]. Few studies have also demonstrated the link between IBS and gut mycobiota. Symptoms of IBS (e.g., diarrhea) are related to *Candida* species overgrowth in patients receiving antibiotic therapy or triggered by *Candida* products [143, 144]. However, more studies should be conducted to reveal the causal relationship between gut mycobiota and those intestinal diseases.

As discussed above, the mucosal immune system may mediate the influence of intestinal fungi on the pathogenesis of intestinal diseases [145, 146]. Especially, intestinal epithelial cells, their resident immune cells [62, 71, 147, 148], the mesenteric lymphatic system [149], cytokines [12], antibodies [71], and the aforementioned fungal metabolites, all may play significant roles in the pathogenesis of intestinal diseases. Notably, the direct or indirect effects of gut mycobiota on intestinal diseases also stimulate further investigations about the mycobiota composition and means of modulating its diversity.

### Gut-brain axis and gut mycobiota

**Fig. 2:**
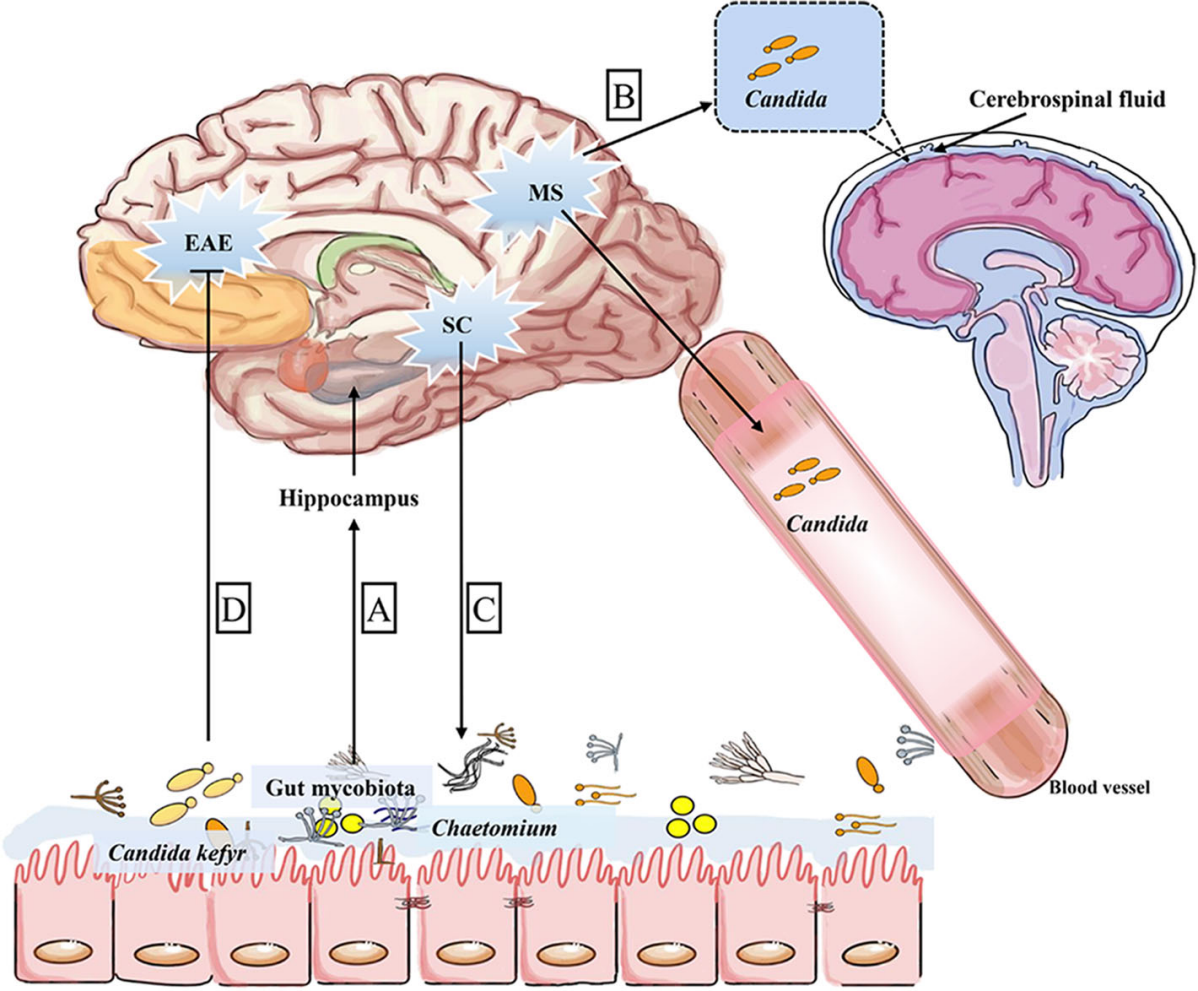
Potential mechanism of gut mycrobiota in gut-brain axis. (**a**) Gut mycobiota modulates the expression of genes in kynurenine pathway and associated micro-RNAs (miRNAs) in the hippocampus in a sex-dependent manner. (**b**) In MS, high *Candida* spp. burden is detected in peripheral blood and cerebrospinal fluid. (**c**) Patients with SC have a changed gut mycobiota alpha diversity and higher abundance of *Chaetomium*. (**d**) *Candida kefyr* supplement in gut is beneficial to alleviate EAE. MS, multiple sclerosis; SC, schizophrenia; EAE, experimental autoimmune encephalomyelitis. Arrows represent activation and horizontal lines represent suppression

Evidence of a plausible gut microbiota-brain axis mainly includes the following: (1) gut microbiota affects the brain via vagus nerve, cytokines, and their metabolic products such as tryptophan, GABA, and acetylcholine; (2) hypothalamus-pituitary-adrenal (HPA) axis plays a core role in communication of gut microbiota and the brain; and (3) gut-brain-microbiota axis provides a new treatment target for depression, autism, and Parkinson disease [150–155]. A recent study revealed that mycobiota regulates expression of genes in the kynurenine pathway and associated micro-RNAs (miRNAs) in the hippocampus in a sex-depend manner [156], suggesting a possible gut mycobiota-brain axis [5]. *Candida kefyr* (also termed as *Candida pseudotropicalis*) is beneficial to alleviate experimental autoimmune encephalomyelitis in C57BL/6 mice [58, 157]. In multiple sclerosis (MS) patients, *Candida* spp. is detected in peripheral blood and in the cerebrospinal fluid [128]. In a cross-sectional study in 10 patients with schizophrenia (SCs) and 16 healthy controls (HCs), the composition and alpha diversity of gut mycobiota changed in SCs, showing enrichment of *Chaetomium* in SCs [129]. The aforementioned findings indicate that *Candida* species might have a significant role in mediating gut mycobiome-brain interaction. As a result, gut mycobiota may hold a significant position in mental diseases (Fig. 2). Although the cross-sectional study had a limited sample size, it emphasized that gut fungi may contribute to the progression of mental diseases. However, we still need to reveal the underlying mechanisms by which gut mycobiota affects the brain, and whether gut fungi communicate through neurotransmitters. Moreover, there is still a clear gap (e.g., various sequencing assays) in the scientific literature regarding fungal involvement in neurological diseases, and some approaches like metagenomics should be employed.

### Gut-lung axis and gut mycobiota

Asthma is a typical chronic allergic airway disease (AAD) regarded as Th2-related disease. According to eosinophil and neutrophil proportion, asthma has been divided into different inflammatory subtypes [158]. In addition to Th2 cells, Th1, Th9, Th17, NKT, CD8^+^ T, and Treg cells are also involved in different types of asthma [159–163]. Many components of fungal cell walls are known to be allergens for asthma, so it is not surprising that fungal disorders are linked to asthma [164, 165]. Notably, the dysbiosis of intestinal fungi caused by oral administration of fluconazole deteriorates lung AAD induced by house dust mite (HDM) [11]. In mice without gut mycobiota, fluconazole treatment had no influence on AAD, indicating that experimentally induced AAD is associated with dysbiosis of gut mycobiota. The underlying mechanism is that fluconazole-induced dysbiosis of gut mycobiota affects the macrophage function, and neutrophil and eosinophil infiltration [11], and Syk-mediated CX3CR^+^ macrophage activation promotes Th2 cell-related responses in the lung, thus participating in the aggravation of AAD [11]. Antimycotics may trigger the expansion of some filamentous fungi (e.g., *Wallemia mellicola*, *Aspergillus amstelodami*, and *Epicoccum nigrum*) to deteriorate AAD through supporting type 2-associated immune responses in the lung [114]. Except for antimycotic-induced gut fungal dysbiosis, antibiotic-induced bacterial disruption also causes *Candida* spp*.* overgrowth and Candida-derived PGE2 overproduction to promote M2 polarization of alveolar macrophages, thus aggravating allergic airway inflammation [105]. Moreover, in asthma, fungal dysbiosis is more significant than bacterial, indicating that fungal disturbance may become ideal marker for asthma detection [164, 166].

**Fig. 3:**
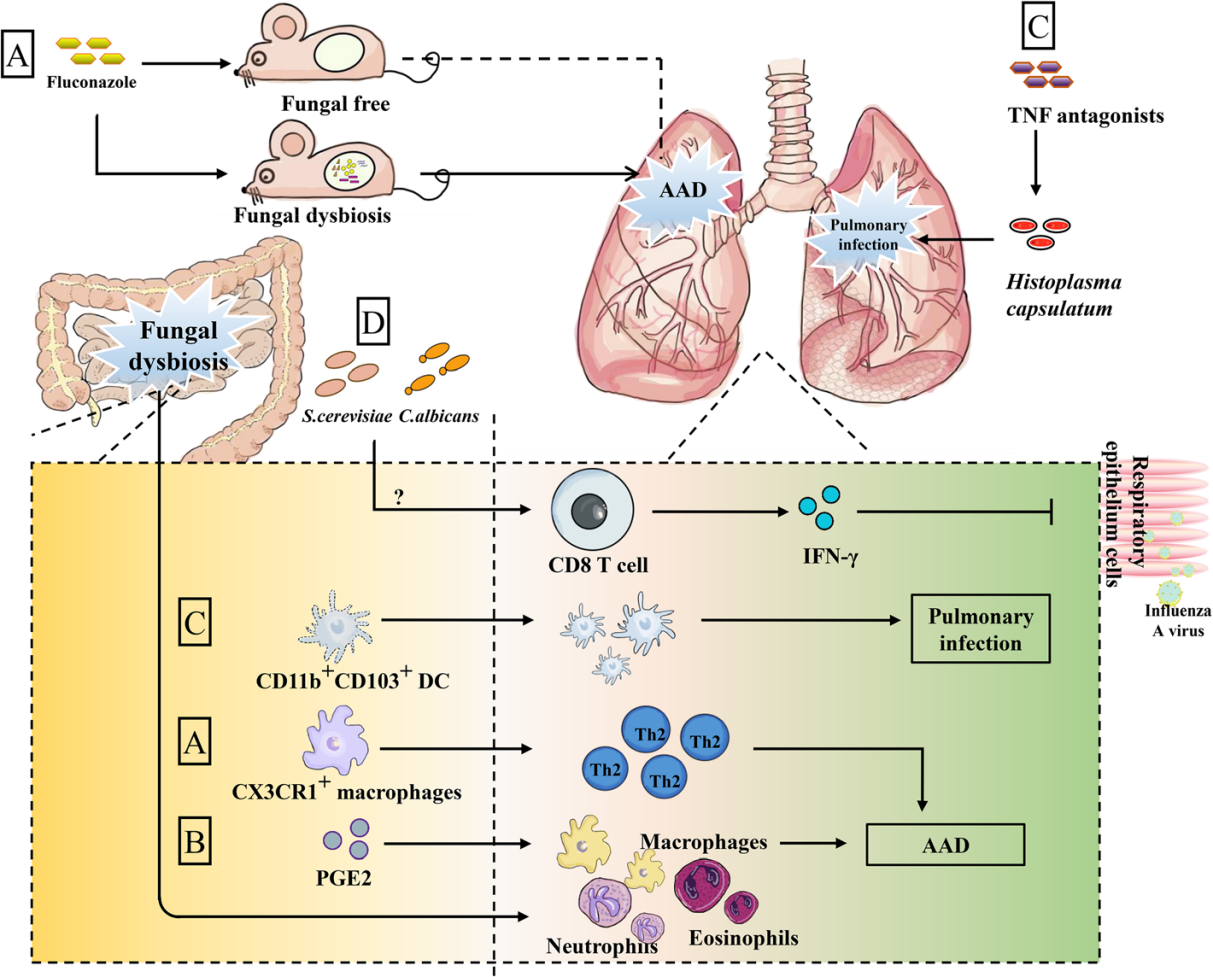
Potential mechanism of gut mycrobiota in gut-lung axis. (**a**) Intestinal fungal dysbiosis caused by fluconazole is sufficient to deteriorate lung AAD induced by house dust mite (HDM) but has no influence in mice without gut mycobiota. Fluconazole-induced dysbiosis of gut mycobiota stimulates intestinal CX3CR^+^ macrophages, leading to Th2 amplification accompanied with lung infiltration by macrophages, neutrophils, and eosinophils. (**b**) Also, gut fungus-induced PGE_2_ promotes M2 polarization of alveolar macrophages to aggravate AAD. (**c**) TNF antagonists enhance the susceptibility of *Histoplasma capsulatum-*induced pulmonary infection, during which intestinal specific CD11b^+^CD103^+^ DCs migrate and augment in the lung to enhance pulmonary infection. (**d**) Intestinal colonization by *C. albicans* and *S. cerevisiae* triggers viral-specific CD8 T cells in the lung (with unknown reasons) and production of IFN-γ, ultimately, preventing the influenza virus from invading the respiratory epithelial cells. AAD, allergic airway diseases; PGE_2_, Prostaglandin E2; TNF, tumor necrosis factor; DCs, dendritic cells. Arrows represent activation and horizontal lines represent suppression

Cystic fibrosis (CF) may be also linked to fungal infections (*Candida* spp*.* are persistent colonizers) [149]. Intestinal colonization of *C. albicans* or *S. cerevisiae* is sufficient to overturn lethal phenomenon caused by influenza A virus [167], which primarily invades the respiratory epithelium cells [168]. Gut immune cells may directly deliver signals about dysbiosis of intestinal mycobiota to the lung [123] (Fig. 3). Moreover, gut microbiota migration potential to the lung has been shown [169]. Consequently, it remains to clarify whether migration of immune cells or the direct migration of gut fungi is related to the gut-lung axis. Also, it is interesting to know whether dysbiosis of gut fungi has different effects on different subtypes of asthma. Furthermore, we also found that fluconazole-induced dysbiosis of gut mycobiota exacerbates the infectious pneumonia (unpublished data), but it is still unknown whether lung neutrophils and/or macrophages mediate the above process. Most intriguingly, the influence of intestinal fungi on commensal fungi in the respiratory tract, and the involvement of respiratory tract fungi on the intestinal fungi-mediated gut-lung axis remain to uncover. Collectively, gut commensal fungi-primed immune cells may be recruited to the lung and may serve as an underappreciated factor affecting the pathogenesis of inflammatory airway diseases (asthma in particular) involved in gut fungal dysbiosis.

### Gut-liver axis and gut mycobiota

**Fig. 4:**
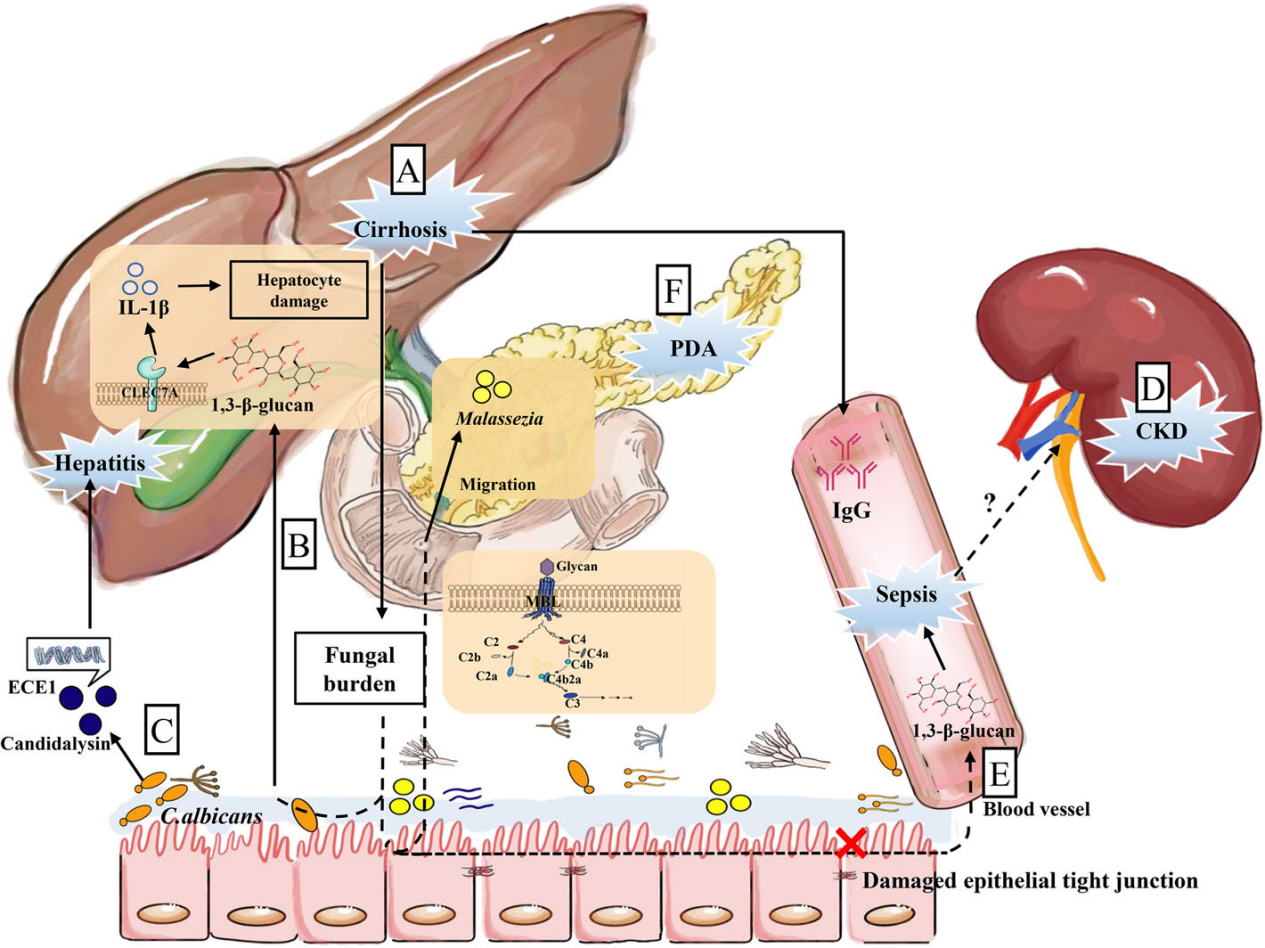
Potential mechanism of gut mycrobiota in gut-liver axis, pancreatic diseases, and gut-kidney axis. (**a**) Patients with liver cirrhosis have a high gut fungal burden and high serum IgG. (**b**) The increased gut fungal burden comes with translocation of 1,3-β-D-glucan (BG). Then, BG is recognized by C-type lectin-like receptor CLEC7A to induce IL-1β production, aggravating hepatocyte damage. (**c**) Intestinal colonized *C. albicans* secrete candidalysin encoded by ECE1 gene, which is involved in exacerbation of the disease in patients with alcoholic hepatitis. (**d**) Mice with CKD show the change in gut microbiota and damaged epithelial tight junctions, leading to leakage of bacterial or fungal products. (**e**) Gut leakage may promote migration of BG in the serum to aggravate sepsis. (**f**) PDA tumor is highly enriched with *Malassezia* spp. in the pancreas, which results from the migration of gut fungi through the direct link between these two organs via the sphincter of Oddi. Besides, glycan of fungal cells wall-MBL-complement cascade pathway plays an important role in PDA. ECE1, extent of cell elongation 1; BG, 1,3-β-D-glucan; CKD, chronic kidney diseases; PDA, pancreatic ductal adenocarcinoma. Arrows represent activation and horizontal lines represent suppression

The liver is an important detoxification organ and participates in defense responses to gut-derived dangers, known as “gut-liver-axis” [170, 171]. Disruption of intestinal microbiota is closely associated with liver diseases such as steatohepatitis and cirrhosis [170, 172]. For instance, after oral administration of ochratoxin A (OTA, a mycotoxin) for ducklings, the diversity of cecum microbiota is reduced, the abundance of LPS-producting *Bacteroidetes* is increased in both cecum and liver, ultimately, OTA promoted liver inflammation through TLR4-Myd88 pathway [172]. Gut mycobiota may be involved in liver diseases through gut-liver-axis. Namely, mycobiota diversity is increased in primary sclerosing cholangitis patients compare with healthy controls [173]. Patients with liver cirrhosis have high abundance of fungi in the duodenum [124], and alcohol abuse-induced cirrhosis is related to *Candida* overgrowth and has higher serum *S. cerevisiae* IgG antibodies [174]. Notably, kefir is a beneficial therapy for anti-alcoholic fatty liver diseases by targeting gut mycobiota [175]. Furthermore, feeding mice with ethanol increases the total intestinal fungal burden, translocation of fungal cell products (e.g., 1,3-β-glucan), and hepatocyte damage [174]. Patients with alcoholic hepatitis show higher percentage of individuals carrying genes for candidalysin (a metabolite from *C. albicans*), and candidalysin treatment exacerbates ethanol-induced liver disease in mice [176]. Thus, gut-liver axis might exist for gut mycobiota, and it probably has great potential for treating liver disease (Fig. 4). Nevertheless, there are still challenges in the field of gut fungi in liver diseases. The existence of the causal relationship between gut fungi and liver diseases needs further compelling investigations. Also, further studies should explore whether the specific fungi isolated from the gut determine the pathogenesis of liver diseases.

### Gut-kidney axis and gut mycobiota

Gut-kidney axis may be a distal target of gut microbiota [177]. Noteworthy, the change of gut microbiota in mice with chronic kidney disease (CKD) is associated with the damaged epithelial tight junctions [178], and bacterial products might leak through intestinal barrier to activate immune responses [177]. Similarly, *Candida* colonization in the gut of patients in intensive-care unit is a susceptibility factor for candidaemia [125]. *C. albicans*-administration in mice might worsen sepsis because of higher serum 1,3-β-D-glucan (BG), altered intestinal microbiota, and production of inflammatory factors [126]. Both studies suggested that intestinal colonization of *C. albicans* may be related to sepsis, and gut leakage may promote migration of BG to aggravate disease process. In ICR mice with *Candida*-disseminated infection, the kidneys have the highest fungal burden [179], suggesting that they may play an important role in circulating fungal infection, but the mechanism remains to be explored. Unfortunately, abundant changes of certain fungi in renal diseases and the presumed BG leakage cannot really explain the existence of the fungal gut-kidney axis. Likewise, investigations of gut mycobiota imbalance direct the pathogenesis of renal diseases are quite limited, thus the fungal gut-kidney axis is speculated to be feasible (Fig. 4). It is worth exploring whether there are other links between gut mycobiota and kidney.

### Gut-pancreas axis and gut mycobiota

Pancreatic beta cells are associated with the pathogenesis of type 1 diabetes mellitus (T1DM) [180–183]. Intriguingly, patients with T1DM and type 2 diabetes mellitus (T2DM) have higher colonization of *C. albicans* compared with healthy controls [184, 185]. Patients with T1DM even have higher diversity of fungal species [186]. These findings indicate that gut fungi may be involved in the pathogenesis of diabetes. However, the evidence about the direct relationship between gut fungi and function of pancreatic beta cells is still limited. Moreover, intestinal commensal bacteria-derived Nod1 ligands (acting as signal molecules) are required for insulin trafficking in pancreatic beta cells [187]. Thus, it would be very interesting to explore whether gut fungi-derived molecules have implications for the function of pancreatic beta cells.

Additionally, in the patients who have pancreatic ductal adenocarcinoma (PDA), bacteria and fungi are markedly increased in pancreas [127, 188]. *Malassezia* spp*.* is highly enriched in PDA in human and mouse, and gut fungi migrate to the pancreas possibly via the sphincter of Oddi [127, 188]. Specifically, mannose-binding lectin (MBL)-deleted (Mbl^−/−^) mice have mild tumor pathogenesis, while recombinant C3a (rC3a) increases pancreatic tumor volume, suggesting that glycan of fungal cell wall-MBL-complement cascade pathway plays an important role in pancreatic diseases (Fig. 4) [127]. However, it is unclear whether dysbiosis of gut mycobiota is the cause of oncogenic progression or just the consequence, and revealing fungal profile for PDA is necessary; therefore, we only propose that Oddi and/or MBL complement cascades might serve as a link in the gut-pancreas axis. Collectively, similar like the gut-kidney axis, the research about fungi-pancreas interaction is still in its early phase.

## Conclusion remarks

Although we generally put more attention on gut bacteriota, it is noteworthy that gut mycobiota also has plenty of potential functions. With the development of technologies for fungal detection, we begin to understand that fungal communities are established immediately after or even before birth. However, it is still unknown how humans become colonized by fungi and whether it is affected by the environment and/or any other factors. Since the establishment of early fungal colonization impacts on later disease status, more direct and continuous research approaches are needed to understand the community of early fungi. The involvement of intestinal fungi in intestinal diseases or diseases in other organs may provide a new window to develop new therapeutic strategies for diseases and provide novel targets for diagnosis. For example, celiac disease usually cannot be diagnosed in a timely manner due to the lack of clinical symptoms, but *Candida* may be involved in the pathogenesis of celiac disease; therefore, if we can establish a direct link between *Candida* and celiac disease, *Candida* could become a diagnostic marker for celiac disease. Nevertheless, more investigations are needed to establish the causality between gut mycobiota and intestinal or extraintestinal diseases. The scientific community eagerly needs well-designed experiments with large number of individuals to compare the alteration of intestinal fungi between patients and healthy controls, and advanced technologies (e.g., metagenomics, metabolomics, and culturomics) to illustrate the underlying mechanisms about how intestinal fungi shape the pathogenesis of diseases. Except for fungus itself, the compounds of gut fungi may have great potential in disease treatments. For example, farnesol is helpful in combinatorial treatments to prevent drug-resistance. Some fungal extracts have antiviral properties, so utilization of fungal compounds might provide possible breakthrough to overcome incurable viral diseases. Previous studies focused mostly on the function of *Candida*, but it should be borne in mind that *Candida* is not the only member of gut fungi and it may not be even the most influential fungus in some diseases. For example, there is no significant difference in *Candida* abundance between patients with schizophrenia and healthy controls [129]. In a word, there is also a long way to find particular fungi uniquely relevant to particular disease, which would qualify as novel marker for diagnosis.

## Data Availability

Data sharing not applicable to this article as no datasets were generated or analyzed during the current study.

## Abbreviations

AA: Arachidonic acid
AAD: Allergic airway diseases
AFLP: Amplified fragment length polymorphism fingerprinting
ASCA: Anti-*Saccharomyces Cerevisiae* Antibody
BA: Blood agar
BG: 1,3-β-D-glucan
BLPs: “Balloon-like” protrusions
CD: Crohn’s disease
CEACAM6: Carcinoembryonic antigen-related cell adhesion molecule 6
CeD: Celiac disease
CF: Cystic fibrosis
CI: *C. albicans* infection
CKD: Chronic kidney disease
CLRs: C-type lectin receptors
DCs: Dendritic cells
DGGE: Denaturing gradient gel electrophoresis
EAE: Experimental autoimmune encephalomyelitis
ECE1: Extent of cell elongation 1
GABA: Gamma-aminobutyric acid
HDM: House dust mite
HPA: Hypothalamus-pituitary-adrenal
HTST: High-throughput sequence technology
HWP1: Hyphal wall protein 1
IBS: Irritable bowel syndrome
iDC: Immature DCs
IgG: Immunoglobulin G
ILCs: Innate lymphoid cells
ITS: Internal transcribed spacer regions
MAPKs: Mitogen-activated protein kinase
MALDI-TOF MS: Matrix-assisted laser desorption ionization time of flight mass spectrometry
MBL: Mannose-binding lectin
MDSCs: Myeloid-derived suppressor cells
MS: Multiple sclerosis
miRNAs: Micro-RNAs
NE: Neutrophil elastase
NET: Neutrophil extracellular traps
NF-κB: Nuclear factor-kappa B
NK: Natural killer
OFRG: Oligonucleotide fingerprinting of ribosomal RNA genes
OTU: Operational taxonomic units
SCs: Schizophrenia patients
PDA: Pancreatic ductal adenocarcinoma
PGs: Prostaglandins
PGE2: Prostaglandin E2
PRRs: Pathogen recognition receptors
QSMs: Quorum-sensing molecules
RALDH+: Retinaldehyde dehydrogenase
rC3a: Recombinant C3a
RFLP: Restriction fragment length polymorphoresis
SCs: Schizophrenia
SCFAs: Short chain fat acids
SDA: Sabouraud dextrose agar
T1DM: Type 1 diabetes mellitus
T2DM: Type 2 diabetes mellitus
TCR: T cell receptor
TG: Transglutaminase
TNF: Tumor necrosis factor
UC: Ulcerative disease
